# Efficacy of Octacalcium Phosphate Collagen Composite for Titanium Dental Implants in Dogs

**DOI:** 10.3390/ma11020229

**Published:** 2018-02-02

**Authors:** Tadashi Kawai, Keiko Matsui, Yushi Ezoe, Fumihiko Kajii, Osamu Suzuki, Tetsu Takahashi, Shinji Kamakura

**Affiliations:** 1 Division of Oral and Maxillofacial Surgery, Tohoku University Graduate School of Dentistry, Sendai 980-8575, Japan; keima@dent.tohoku.ac.jp (K.M.); geka0329@dent.tohoku.ac.jp (Y.E.);tetsu@dent.tohoku.ac.jp (T.T.); 2 Toyobo Co., Ltd., Otsu 520-0292, Japan; Fumihiko_Kajii@toyobo.jp; 3 Division of Bone Regenerative Engineering, Tohoku University Graduate School of Biomedical Engineering, Sendai 980-8574, Japan; kamakura@bme.tohoku.ac.jp; 4 Division of Craniofacial Function Engineering, Tohoku University Graduate School of Dentistry, Sendai 980-8575, Japan; suzuki-o@m.tohoku.ac.jp

**Keywords:** bone substitute, osseointegration, dental implant

## Abstract

Background: Previous studies showed that octacalcium (OCP) collagen composite (OCP/Col) can be used to repair human jaw bone defects without any associated abnormalities. The present study investigated whether OCP/Col could be applied to dental implant treatment using a dog tooth extraction socket model. Methods: The premolars of dogs were extracted; each extraction socket was extended, and titanium dental implants were placed in each socket. OCP/Col was inserted in the space around a titanium dental implant. Autologous bone was used to fill the other sockets, while the untreated socket (i.e., no bone substitute material) served as a control. Three months after the operation, these specimens were analyzed for the osseointegration of each bone substitute material with the surface of the titanium dental implant. Results: In histomorphometric analyses, the peri-implant bone areas (BA%) and bone-implant contact (BIC%) were measured. There was no difference in BA% or BIC% between OCP/Col and autologous bone. Conclusion: These results suggested that OCP/Col could be used for implant treatment as a bone substitute.

## 1. Introduction

Bone augmentation is an important aspect of dental implant therapy for alveolar bone atrophy. Various techniques for augmentation, including sinus floor elevation [1,2], split crest [3,4], and guided bone regeneration [5], are used during dental implant surgery, along with bone-filling materials. Autologous bone grafting is the gold standard for bone defect reconstruction owing to its high osteoconductivity [6,7]. However, it has certain disadvantages, including limited availability and morbidity associated with harvesting bone from a secondary operative site [8,9,10]. Calcium phosphate is used in clinical settings as a bone substitute material [11,12,13]. Although it is abundantly available, it does not have the high osteoconductivity seen with autografts [14]. Hydroxyapatite (HA) [15], β-tricalcium phosphate (β-TCP) [16], and xenogenic grafts, such as bovine bone [17], have been used clinically as bone substitutes. These materials have efficacy for titanium dental implantation and show good osseointegration [15,18,19], although they are inferior to autologous bone in these respects. Many studies have been performed with the aim of improving osteoconductivity by combining these materials [20], or incorporating other factors [21] or mesenchymal stem cells [22]. Today, other groups have reported superior bone regeneration by the mixing of autologous bone and artificial material [23].

Octacalcium phosphate (OCP) has been suggested to be a precursor of biological apatite in bones and teeth [24], and has been detected in porcine enamel [25], human dentin [26], and mouse calvarial bone [27] as an intermediate in the formation of apatite matrices. Previous studies have demonstrated that OCP induces osteoblastic cell differentiation to a greater degree than HA [28,29] and β-TCP [30]. OCP is irreversibly converted to biological apatite crystals under biological conditions [29], and bone formation by implanted OCP has been reported in vivo [29,31]. OCP enhances bone regeneration and is more readily absorbed than HA or β-TCP [32]. OCP and collagen composite (OCP/Col) developed for improved handling showed a greater ability to promote bone regeneration as compared to OCP alone [33]. Previous studies demonstrated that large bone defects in dogs could be reconstructed with OCP/Col [34,35,36,37,38,39], which was completely converted to bone-like apatite and incorporated into newly-formed bone in the defect [34,35,38]. Recent studies reported the first clinical application of OCP/Col in a cystectomy or tooth extraction defect and obtained promising results [40,41]. Another group has also reported the clinical use of OCP granules for the defect after resection of fibrous dysplasia at the mandible or generation of space by sinus floor elevation [42]. The present study investigated whether OCP/Col could be applied to dental implant treatment as a bone substitute, evaluated the osseointegration of newly-formed bone by OCP/Col to titanium dental implants, and compared it with autologous bone.

## 2. Materials and Methods

### 2.1. Animals

Male beagle dogs (age: 18 months, Kitayama Labes Co., Ltd., Ina, Japan) were used for experimentation. The study protocol followed the principles of laboratory animal care, as well as national laws, and was approved by the Animal Research Committee of Tohoku University (approval number: 2015-dental-animal-008). In the experiment facilities of Tohoku University, the temperature and humidity were managed. The laboratory animals were bred using a cage of the experiment facilities. The laboratory animals were managed by a contracted veterinarian, and the laboratory animals engaged in periodic activity and received examination regularly. After experimentation, the animals received a soft diet and had free access to drinking water. We carefully observed the presence of harmful phenomena, such as wound compartment infection, decreased appetite, and death.

### 2.2. Preparation of OCP/Col and Autologous Bone

**Figure 1:**
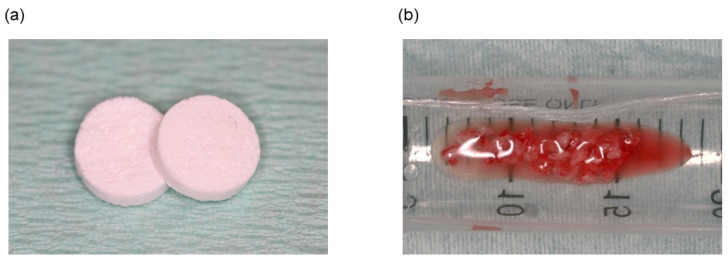
(**a**) Image of OCP/Col discs. The discs were 9 mm in diameter and 1.5 mm thick. (**b**) Autologous bone from each tooth extraction socket.

OCP was prepared by mixing calcium acetate hydrate and sodium phosphate monobasic solutions, as previously described [31]. The precipitates were washed several times with sterile filtered water and then lyophilized. Sieved OCP granules (particle size: 300–500 μm) obtained from dried OCP were sterilized by heating at 120 °C for 2 h. Powder X-ray diffraction (XRD) patterns of the obtained OCP were determined by step-scanning at 0.05° intervals from 3.00° to 60.00°, with Cu Kα X-rays on a diffractometer (Mini Flex; Rigaku Electrical Co., Tokyo, Japan) at 30 kV and 15 mA. The 2θ range included the primary peak (100) reflection of OCP at approximately 4.7°. Collagen was obtained from Nippon Meat Packers collagen PS (Tsukuba, Japan) and a lyophilized powder was prepared from pepsin-digested atelocollagen isolated from porcine dermis. The collagen was dissolved in sterile-filtered water and adjusted to a final concentration of 3% at pH 7.4. OCP/Col was prepared by adding OCP to the concentrated collagen and mixing, yielding an OCP weight percentage of 77%. The OCP/Col mixture was then lyophilized and molded into discs 9 mm in diameter and 1.5 mm thick (Figure 1a) that were subjected to dehydrothermal treatment (150 °C for 24 h) in a vacuum drying oven (DP32; Yamato Scientific, Tokyo, Japan). The OCP/Col was sterilized by electron beam irradiation (15 kGy) before use. The XRD pattern indicated a collapsed and reduced the primary (100) peak with a shift from 4.7° to 5.3° degrees at 2θ, as previously reported [24].

Autologous bone was collected from the extracted tooth socket while extending the socket using forceps (Figure 1b). 

### 2.3. Surgical Procedure

**Figure 2:**
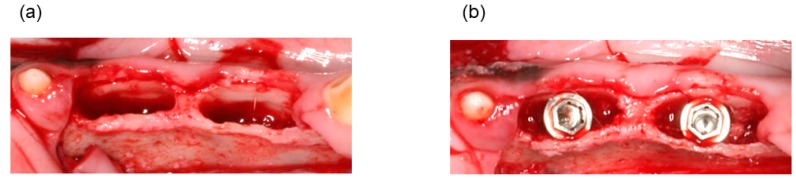

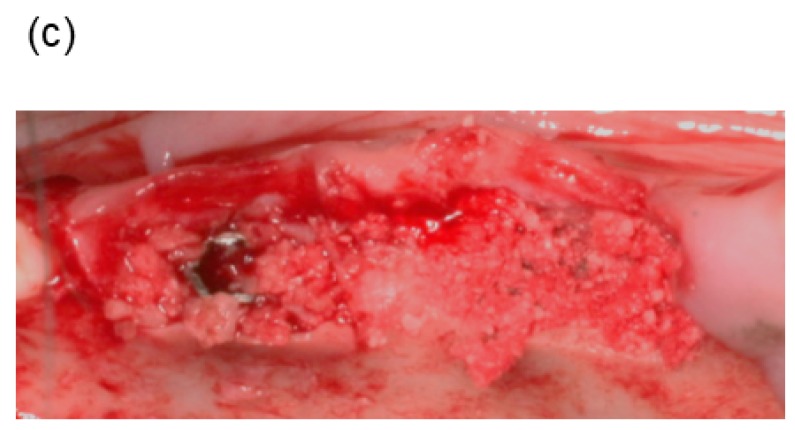
(**a**) Preparation of bone defects. After tooth extraction, the sockets were extended. The depth of the extended socket was at least 5 mm and the mesiodistal width was at least 10 mm. (**b**) Placement of titanium dental implants in each socket. (**c**) Defects were filled with bone substitute material. Left and right sides show autologous bone and OCP/Col implantation, respectively.

Animals were anesthetized by intravenous administration of sodium pentobarbital (25 mg/kg), followed by intramuscular injection of atropine sulfate (0.25 mg) and ketamine hydrochloride (20 mg/kg). After disinfection of the oral cavity, local anesthetic was administered by injecting 2% lidocaine with 1/80,000 epinephrine. A gingival incision was added and a buccal mucoperiosteal flap was formed. After extraction of the second and third premolars of the bilateral mandibles, the four extraction sockets were extended and the alveolar septa were removed using forceps. The depth of the extended tooth extraction socket was at least 5 mm and the mesiodistal width was at least 10 mm (Figure 2a). A titanium dental implant (Brånemark System**^®^** Mk III Groovy, Nobel Biocare Japan K.K., Tokyo, Japan) with an external diameter of 4 mm and a length of 11.5 mm was inserted at the center of each socket (Figure 2b). The OCP/Col, or autologous bone was inserted in the space between the residual socket and the titanium dental implant (Figure 2c). The sixteen sockets of four dogs were used for the experiment, six sockets were used for OCP/Col, six sockets were used for autologous bone, and the remaining four sockets were used as controls. The buccal mucoperiosteal flap was repositioned and sutured. To prevent infection, flomoxef sodium (Flumarin**^®^**, Shionogi and Co., Osaka, Japan) was administered by intravenous drip during the operation, and cefcapene pivoxil hydrochloride hydrate (Flomox**^®^**, Shionogi and Co., Osaka, Japan) was orally administered for three days after the operation. All the dogs were sacrificed three months after the operation with an overdose of sodium pentobarbital (20 mL) administered by intravenous injection. Implants were removed with the surrounding bones and tissue and fixed over four weeks by infiltration with 10% formalin in neutral buffer solution (pH 7.4) at 4 °C.

### 2.4. Radiographic Analysis

Intra-oral radiography was performed immediately after operation using a MAX-DC70 instrument (J. Morita Manufacturing, Kyoto, Japan) with instant films (Hanshin Technical Laboratory, Nishinomiya, Japan) under standardized conditions (60 kV, 10 mA, 0.5 s). Dissected mandibles were subjected to X-ray (FR; Fuji Photo Film, Tokyo, Japan) microradiography (Softex CMBW-2; Softex, Kanagawa, Japan) under standardized conditions (40 kV, 5 mA, 20 s).

### 2.5. Contact Microradiography, Histological, and Histomorphometric Analyses

Specimens were fixed in 70% ethanol, stained with Villanueva bone stain, dehydrated in a graded series of ethanol, and embedded in methyl methacrylate. The specimens were then sectioned using a low-speed saw (Isomet 5000; Buehler, Lake Bluff, IL, USA) with a diamond-wafering blade. Sections were mounted on plastic slides and were ground and polished until they were 200–300 µm thick. Contact microradiography of undecalcified sections was performed with a Softex M-60 microradiography unit (Softex Co., Ltd., Ebina, Kanagawa, Japan) for 120 s exposures, at 45 kV and 1.5 mA. The sections were then observed with a photomicroscope (DFC290HD; Leica Microsystems, Tokyo, Japan). The percent peri-implant bone area (BA%) was measured as the area occupied by the bone tissue within the threads of the implant and was expressed as a percentage of the total area of the implant threads. The percent bone-implant contact (BIC%) was measured as the length of bone in direct contact with the implant surface within the threads and was expressed as a percentage of the total perimeter of implant threads. BA% and BIC% were measured in the range of 4 mm from 1 mm under the first thread in digital images of sections using Image J software (National Institute of Health, Bethesda, MD, USA).

### 2.6. Statistical Analysis

Histomorphometric data were analyzed using Excel v.10 software (Microsoft, Redmond, WA, USA). All values were reported as the mean ± standard deviation. Mean differences among groups were compared by one-way analysis of variance; significant differences were further analyzed with the Tukey multiple comparisons post hoc test.

## 3. Results

### 3.1. Clinical Observation

Sixteen implants were placed in four dogs. No adverse events occurred related to the surgical protocol in any specimens. All of the specimens were evaluable in this experiment (OCP/Col = 6, autologous bone = 6, Untreated = 4).

### 3.2. Radiographic Observations

**Figure 3:**
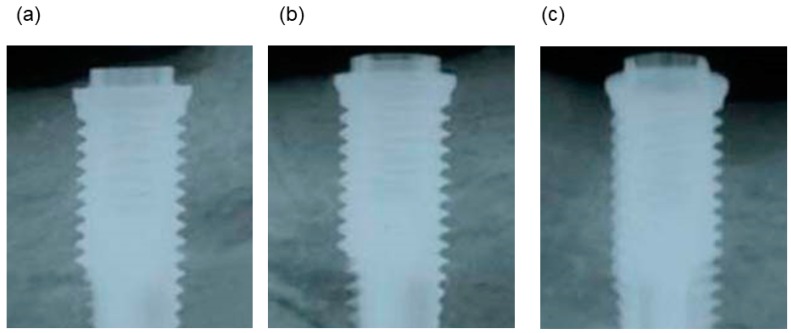
X-ray images of specimens three months after operation. (**a**) OCP/Col. (**b**) Autologous bone. (**c**) Untreated.

Given that OCP and OCP/Col have low radiopacity under standard X-ray conditions, the radiographic signal in the space between the host bone and titanium dental implant was negligible for OCP/Col immediately after operation. In contrast, autologous bone exhibited radiopacity. The untreated region showed the original defect around the implant. At three months after the operation, OCP/Col and autologous bone had converted to hard tissue with the same radiopacity as the surrounding bone (Figure 3a,b), whereas the untreated defect was unaltered (Figure 3c).

### 3.3. Contact Microradiography and Histological Observations

**Figure 4:**
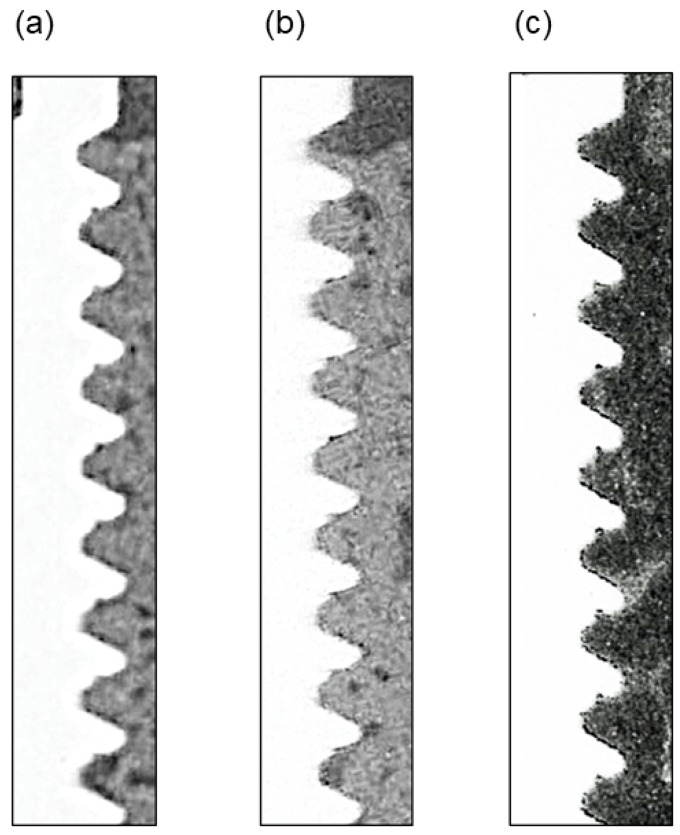
Contact microradiography of specimens. (**a**) OCP/Col. (**b**) Autologous bone. (**c**) Untreated. The white part on the left side of the image is the implant body. The gray-colored part shows new bone. There is almost no gap between the titanium implant body and the newly-formed bone. Minimal bone regeneration was observed in the untreated defect.

**Figure 5:**
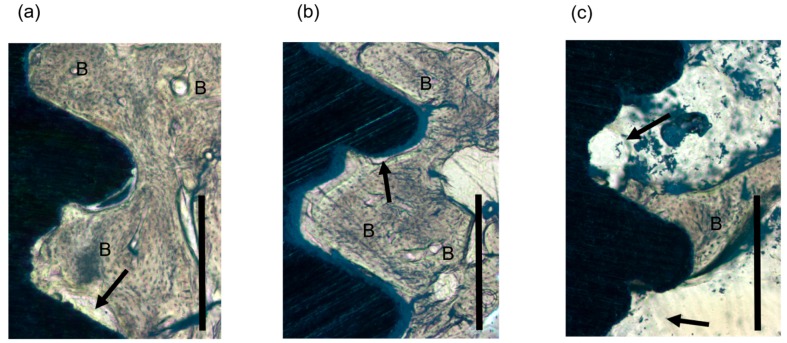
Histological analysis of specimens. (**a**) OCP/Col. (**b**) Autologous bone. (**c**) Untreated. Mature bone was stained green and the osteoid was stained red in newly-formed bone of each sections. Arrows show the area of non-bone contact area. B = newly-formed bone, bars = 200 μm.

Contact microradiography images are shown in Figure 4. There is almost no gap between the titanium implant body and the newly-formed bone. Some bone marrow cavities were confirmed in the newly-formed bone. In histological sections, normal bone tissue was present in the space implanted with OCP/Col, and we could not confirm the presence of remaining OCP in the newly-formed bone (Figure 5a), which had spread within the threads to make contact with the surface of the dental implant. Similar observations were made for the defect implanted with autologous bone (Figure 5b). There was little newly-formed bone in the space around the implant for the untreated defect (Figure 5c). Osteoid stained red was uniformly mixed in mature bone stained green in OCP/Col or autologous bone groups.

### 3.4. Histomorphometric Findings

**Figure 6:**
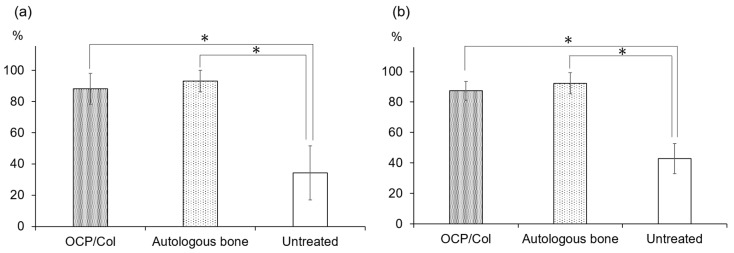
Histomorphometric analysis of specimens. (**a**) BA% and (**b**) BIC% are shown. * *p* < 0.01.

The BA% of OCP/Col, autologous bone, and the untreated defect was 88.28% ± 9.98%, 93.27% ± 6.90%, and 34.32% ± 17.25%, respectively (Figure 6a). Although, the values for OCP/Col and autologous bone differed with respect to the untreated control, there was no difference among them. The BIC% of OCP/Col, autologous bone, and the untreated defect was 87.35% ± 6.28%, 92.30% ± 6.98%, and 42.95% ± 9.93%, respectively (Figure 6b). Each of the two bone substitutes showed a significant difference with respect to the control. There was no difference in BIC% between OCP/Col and autologous bone.

## 4. Discussion

Dental implant experiments using dogs are often chosen because the jaw of dogs resembles that of humans [43]. This study evaluated osseointegration and the efficacy of bone substitutes for a titanium dental implant. 

There are sterilization treatments at 120 °C for 2 h in OCP preparation, our previous study showed that such heating does not affect physical properties of OCP granules, such as the crystalline structure or specific surface area [44], although it has been reported that temperatures over 100 °C caused the OCP structure to collapse as a result of dehydration [45,46].

Radiographic analyses revealed the conversion of OCP/Col into a radiopaque hard tissue three months after operation. Autologous bone also showed suitable radiographic results at three months. Contact microradiography revealed strong contact of hard tissue thought to be newly-formed bone with the titanium surface on implantation with OCP/Col and autologous bone. The histological analysis revealed that when the autologous bone graft was used as a bone substitute, most of the bone defect around the dental implant was filled with newly-formed bone, which also covered most of the implant surface. Mature bone and osteoid were complicatedly mixed. The maturation of newly-formed bone by OCP/Col has been thought to be done by six months [35]. Since osteoid was confirmed homogeneously in newly-formed bone, it was thought that it was still in the maturation process after three months. Residual material could not be identified by Villanueva bone staining with undecalcified sections in the current study. The remaining OCP granules were confirmed in the experiment with rat or dog skull defects [33,35]. However, it was not easy to identify the remaining OCP granules in the OCP/Col implantation experiment in the canine jaw defect area in previous studies [36,37]. It seems that the absorbability of OCP is high in the jaw compared to the skull.

The BA% and BIC% (93.27 ± 6.90% and 92.30 ± 6.98%, respectively) indicated high osteoconductivity and affinity of the autologous bone graft for the dental implant. OCP/Col was also able to repair the bone defect around the dental implant: the bone that was newly-formed by OCP/Col showed similar histological features as for autologous bone and nearly covered the implant surface, implying that OCP/Col has equally high efficacy for the implant. Although the BA% and BIC% for OCP/Col (88.28 ± 9.98% and 87.35 ± 6.28%, respectively) were lower than the corresponding values for autologous bone, this difference was not statistically significant, whereas OCP/Col and autologous bone had markedly higher BA% and BIC% values than the untreated defect (34.32 ± 17.25% and 42.95 ± 9.93%). Tukey post hoc test was chosen to confirm the significant difference between all groups, including the OCP/Col group and autologous bone group. In the present study there was no significant difference between OCP/Col and autologous bone.

In this study, since the evaluation of the affinity between newly-formed bone by OCP/Col and a titanium implant was completed, the experimental period was only three months. In order to evaluate the stability of dental implantation using OCP/Col, it is necessary to observe the situation over a longer period of time. However, this study showed the efficacy of OCP/Col in the early stage of dental implant treatment.

## 5. Conclusions

In this study, the newly-formed bone by OCP/Col was in contact with the titanium implant surface, and there was no significant difference from autologous bone. Therefore, it was suggested that OCP/Col could be used for implant treatment as a bone substitute.

## 6. Patents

Shinji Kamakura and Osamu Suzuki obtained a patent for OCP/Col in Japan (#5046511).
